# Effect of varying doses of dexmedetomidine added to dexamethasone-enhanced TAPB for post-cesarean pain management

**DOI:** 10.3389/fmed.2025.1593574

**Published:** 2025-07-29

**Authors:** Yang Gu, Fan Yang, Jiamin Bao, Fa Wang, Biyun Tian, Hui Sun, Ningkang Li, Qingshan Ye

**Affiliations:** ^1^Department of Anesthesiology, Ningxia Medical University, Yinchuan, China; ^2^Department of Anesthesiology, People’s Hospital of Ningxia Hui Autonomous Region, Ningxia Medical University, Yinchuan, Ningxia, China; ^3^Department of Anesthesiology, General Hospital of Ningxia Medical University, Yinchuan, China

**Keywords:** dexmedetomidine, dexamethasone, nerve blocks, pain, postoperative, cesarean sections

## Abstract

**Background:**

The management of post-cesarean pain exhibits considerable variation across different regions and hospitals, with a prevalent tendency to utilize opioid medications as the primary analgesic approach. This study investigates the impact of different doses of dexmedetomidine combined with dexamethasone as an adjunct to transversus abdominis plane block (TAPB) on the analgesic efficacy and quality of recovery following cesarean section.

**Methods:**

In this prospective randomized clinical trial, 90 patients scheduled for cesarean section were randomly assigned in a 1:1:1 ratio to receive postoperative TAPB with one of three solutions: 8 mg dexamethasone with 0.375% ropivacaine (Group C), 0.5 μg/kg dexmedetomidine with 8 mg dexamethasone and 0.375% ropivacaine (Group D1), or 1 μg/kg dexmedetomidine with 8 mg dexamethasone and 0.375% ropivacaine (Group D2). The primary outcome measures were the VAS scores for rest and movement at 6, 12, 24, and 48 h post TAPB, as well as the incidence of moderate to severe pain.

**Results:**

Postoperative VAS scores demonstrated distinct patterns between rest and dynamic pain. At rest, no significant differences were observed among groups C, D1, and D2 at any time point (6–48 h; all *p* > 0.05). For dynamic pain, group C exhibited higher median scores than D1 and D2 at 12 h [3.00 (IQR 2.00–4.00) vs. 1.00 (1.00–3.00), median difference 1.00 (95% CI 1.00–2.00); *p* = 0.001; vs. 2.00 (1.00–3.00), difference 1.00 (0.00–2.00); *p* = 0.003] and 24 h [4.00 (3.00–4.00) vs. D1: 3.00 (2.00–3.00), difference 1.00 (0.00–1.00); *p* < 0.001; vs. D2: 2.00 (2.00–3.00), difference 1.00 (1.00–2.00); *p* = 0.009]. By 48 h, D2 showed the lowest dynamic pain scores [1.00 (1.00–2.00) vs. C: 3.00 (2.00–3.00); difference 1.00 (1.00–1.00); *p* = 0.001]. Moderate-to-severe dynamic pain incidence differed significantly at 12 h (C: 26.7%; D1: 13.3%; D2: 3.3%; *p* = 0.04) and peaked in group C at 24 h [53.3% vs. D1: 13.3% (risk ratio 7.43, 95% CI 2.08–26.55; *p* = 0.002) and D2: 10.0% (risk ratio 10.29, 2.56–41.37; *p* = 0.001)]. No intergroup differences were observed for resting pain or dynamic pain at 48 h. Groups D1 and D2 showed no significant differences in outcomes at any time point.

**Conclusion:**

Adding dexmedetomidine and dexamethasone to ropivacaine for TAPB can improve post-cesarean section pain conditions.

**Clinical trial registration:**

https://clinicaltrials.gov/, ChiCTR2400081531.

## Introduction

Despite the development and publication of post-cesarean pain management guidelines by numerous countries and regions worldwide, significant variations persist in actual pain management practices both between nations and across different regions within the same country (1–5). Current research investigations have found that a significant number of patients who undergo cesarean sections still report high pain scores postoperatively (6, 7). This phenomenon strongly suggests that this patient population may generally be facing an issue of suboptimal analgesic efficacy.

Achieving adequate and effective analgesia post-cesarean section is of critical importance (8). It not only facilitates early mobilization for postpartum women, aiding in the recovery of bodily functions, but also promotes effective infant care, including successful breastfeeding initiation and fostering a deep emotional connection between mother and baby (9). Conversely, inadequate management of acute pain after cesarean sections may lead to chronic pain issues and even contribute to post-traumatic stress disorder in mothers, resulting in long-term and serious adverse effects on their mental and physical health (10).

Ultrasound-guided transversus abdominis plane block (TAPB) is recommended as an essential component of the multimodal pain management protocol for cesarean sections (11, 12). In recent years, some studies have shown that the combined use of dexmedetomidine and dexamethasone in the TAPB can effectively prolong the duration of analgesia and significantly improve postoperative pain perception in certain surgical patients (13). However, when this combined drug regimen is applied to different types of nerve blocks and corresponding surgeries, the duration of the blocks shows certain variations, ranging approximately from 13 to 25 h (14–18). Given that the postoperative pain curve for obstetric patients typically peaks within 24 h after surgery (19), the potential application of dexmedetomidine and dexamethasone in the TAPB for cesarean section patients is likely to benefit these patients. Unfortunately, relevant research data in this area are still relatively scarce.

We hypothesize that the adjunct use of two drugs with different mechanisms of action in the TAPB procedure may optimize pain relief after cesarean section. Therefore, this study aims to verify whether the combination of different doses of dexmedetomidine and dexamethasone, used as adjuvant drugs in the transversus abdominis plane block (TAPB), can effectively improve the quality of analgesia after cesarean section.

## Materials and methods

This study was approved by the Ethics Committee of the People’s Hospital of Ningxia Hui Autonomous Region (Approval No. 2024-LL-005), and informed written consent was obtained from all subjects participating in the trial. The trial was registered before patient enrollment at the Chinese Clinical Trial Registry (ChiCTR2400081531; registration date: March 4, 2024). The study was conducted from March 6 to September 20, 2024, with the first patient enrolled on the commencement date (March 6, 2024).

This study was a prospective, randomized, double-blind controlled trial. The trial aims to include 90 patients aged between 20 and 42 years, with an American Society of Anesthesiologists (ASA) physical status classification of II or III, a body mass index (BMI) ranging from 18.0 to 35.0 kg/m^2^, at 37–42 weeks of gestation, who were undergoing cesarean section surgery under spinal anesthesia. This study excluded patients with the following conditions: contraindications to neuraxial anesthesia, including a platelet count<70 × 10^9/L; significant coagulation dysfunction; severe spinal deformity; infection at the puncture site; inability to understand or cooperate with the Visual Analog Scale (VAS) for pain assessment; allergic reactions to the medications used in the study; presence of bradycardia or atrioventricular block; preoperative use of sedatives or hypnotics; patients actively using analgesics for acute/chronic pain disorders, or those with active untreated pain disorders; and a history of neurological or psychiatric disorders. After the commencement of this study, there were no significant changes made to the experimental methods.

### Randomization method and blinding

Patients were randomly allocated to one of three groups using a computer-generated randomization sequence with a 1:1:1 ratio, which was concealed in sequentially numbered, sealed, opaque envelopes (numbered 1–90). The physicians administering anesthesia were aware of the group assignments, while the parturients and the anesthesiologists evaluating the outcome indicators were blinded.

### Intraoperative and postoperative procedures

Routine monitoring of the patient’s vital signs was performed after the patient entered the operating room. An 18–20 G intravenous cannula was used to establish peripheral vascular access, and compound sodium chloride solution was administered at a rate of 20 mL·kg^−1^·h^−1^. The patient was positioned in the left lateral decubitus position, and the puncture site at the L2-3 intervertebral space was identified. The skin was disinfected and draped, and local anesthesia was administered. A 9 G guide needle was used for the initial puncture, after which a 25 G spinal needle was employed to puncture the dura mater. Upon entering the subarachnoid space, cerebrospinal fluid was observed to flow smoothly, and no blood was detected during aspiration. Subsequently, 3 mL of a mixed solution containing 0.5% bupivacaine and 50 μg of fentanyl was slowly injected. After confirming the level of anesthesia, both the introducer needle and the spinal needle were removed simultaneously. The parturient was then placed in a supine position, and the level of anesthesia was adjusted to T6.

Following the completion of surgery, a TAP block was performed under the guidance of an ultrasound device (brand: Mindray TE7, probe model: L14-6Ns, frequency: 7–13 MHz). The anesthesiologist wore sterile gloves, placed a sterile plastic sheath over the transducer, and disinfected the skin with a 0.5% chlorhexidine solution. The transducer was positioned vertically along the midaxillary line, approximately at the horizontal level of the T10 dermatome. When the external oblique, internal oblique, transversus abdominis muscles, and the peritoneum were clearly visualized, the in-plane needle insertion technique was used to advance a 21 G, 100 mm nerve block needle (produced by Pajunk GmbH, Geisingen, Germany) into the area between the internal oblique and transversus abdominis muscles. After observing no blood upon aspiration, each patient was injected with a 20 mL mixture into each side of the transversus abdominis plane. The formulation of the 20 mL mixture for patients in each group was as follows: Group C contained 8 mg of dexamethasone, 75 mg of ropivacaine, and normal saline; Group D1 contained 8 mg of dexamethasone, 75 mg of ropivacaine, and 0.5 μg/kg of dexmedetomidine; Group D2 contained 8 mg of dexamethasone, 75 mg of ropivacaine, and 1 μg/kg of dexmedetomidine. During the administration of local anesthetics, signs indicative of compression of the transversus abdominis muscle may manifest as a progressive enlargement of the corresponding hypoechoic area in ultrasonographic imaging. After leaving the operating room, all patients were administered a disposable infusion pump (CBI + PCA) to implement the intravenous analgesia protocol. The analgesic medication is formulated by combining 100 μg of sufentanil, 4 mg of hydromorphone, and 8 mg of tropisetron with normal saline, resulting in a total volume of 110 mL. The infusion pump parameters were set as follows: Continuous Background Infusion (CBI) rate 2 mL/h, Patient-Controlled Analgesia (PCA) bolus dose 0.5 mL, lockout interval 15 min. Patients were instructed to press the analgesic pump for self-administered pain relief when they felt significant pain. If, after pressing the analgesic pump, the pain was still unbearable, they were advised to inform the ward nurse. The attending physician administered two Lofen Codeine Tablets (0.2 g*12.5 mg) as rescue analgesia. All anesthetic procedures were performed by the same anesthesiologist.

### Outcome measures

Primary Outcome Measures: Static and dynamic VAS scores at 6, 12, 24, and 48 h following TAPB completion (the definition of a static VAS score is the pain rating reported by a patient while at rest; the dynamic VAS score is defined as the score during knee and hip joint flexion; the VAS pain score is defined as 0 cm = no pain, 10 cm = the worst imaginable pain) and the incidence of moderate to severe postoperative pain [moderate to severe postoperative pain is defined as a VAS score>3 (20)].

Secondary Outcome Measures: The proportion of patients who first requested rescue analgesia within the first 24 h postoperatively and between 24 and 72 h; The duration of sensory blocked following TAPB (The duration of sensory nerve blocked was evaluated using two standardized sensory assessments: the pinprick test and cold sensitivity test. Specifically, the duration of sensory nerve blocked was defined as the time from the completion of the TAPB until the patient could again perceive the cold sensation of an alcohol swab (temperature discrimination) or the pain from a needle prick (mechanical discrimination). Standardized sensory assessments were conducted at 12-h intervals during the postoperative period. During the patient’s hospitalization (at postoperative hours 6, 12, 24, 48, and 72), bedside assessments were conducted by a uniformly trained research team member (an anesthesiologist) using the pinprick test and cold sensitivity test. Following discharge, data collection continued via standardized telephone follow-up. Patients were instructed to perform self-reporting using the same assessment methods employed by the research team. Importantly, detailed training on the correct execution of both the pinprick test and cold sensitivity test was provided to each participant during the informed consent process. Furthermore, at every bedside assessment during the hospitalization period, the research personnel dedicated to postoperative evaluations provided on-site reinforcement of the patient’s self-assessment skills); The incidence of postoperative nausea and vomiting (0–6 h and 6–24 h); The time to postoperative gastrointestinal recovery (defined as the time interval between preoperative fasting initiation (last oral water intake) and the first documented passage of flatus in the postoperative period) and the score of the Obstetric Quality of Recovery-10 (ObsQoR-10) (All ObsQoR-10 assessments were performed by a single trained anesthesiologist on postoperative days 1, 3, and 7. Patients were awake and cooperative throughout the entire evaluation process. Assessments were conducted using a paper-based questionnaire, with the anesthesiologist reading each item aloud and recording the patients’ responses accordingly. If the patient remained hospitalized, assessments took place via face-to-face interviews at the bedside; if the patient had already been discharged, data collection was completed through standardized telephone follow-up).

### Sample size calculation

Based on the preliminary results from 12 cases in each group, the VAS score at 12 h postoperatively was used to estimate the sample size. The mean (SD) of the VAS scores at 12 h postoperatively for the three groups was as follows: Group C: 3.50 (1.44), Group D1: 2.58 (1.31), and Group D2: 2.17 (1.12) points. Using the sample size calculation software NCSS PASS (version 15), with a power of 1-*β* = 0.9 and a significance level of *α* = 0.05, it was calculated that the sample size required for each group is 25 cases. Considering a potential dropout rate of 20%, a total of 90 patients were ultimately included across the three groups. The data analyzed in this study did not include information from the preliminary experiment.

### Statistical analysis

Statistical tests were conducted using SPSS 17.0 (SPSS Inc., Chicago, IL, United States). The Shapiro–Wilk test was applied to continuous variables for normality testing. Continuous variables that were normally distributed were represented as mean (SD), while non-normally distributed continuous variables were represented as median (IQR). Continuous variables included VAS pain scores, duration of sensory blocked, gastrointestinal recovery time, ObsQoR-10 scale scores, and patient characteristics (age, height, weight). Continuous variables that conformed to a normal distribution among the three groups were analyzed using one-way analysis of variance (ANOVA). When ANOVA indicated statistical significance, that is, *p* < 0.05, the least significant difference (LSD) method was used for pairwise comparisons. Variables that did not conform to a normal distribution were analyzed using the Kruskal-Wallis non-parametric test, and pairwise comparisons were made using the Mann–Whitney test. The median difference (and 95% CI) was calculated using the Hodges-Lehmann estimators. Categorical variables were represented as counts (percentages), and comparisons between groups were made using the Chi-square test or Fisher’s exact test. Categorical variables included the proportion of first request of rescue analgesia, the incidence of nausea, vomiting, moderate to severe pain, and the number of primiparous women. All statistical tests were two-sided, and a *p* < 0.05 was considered significant. In multiple comparisons, the significance level for the pairwise comparison test was adjusted to 0.05/3.

## Results

### Baseline data

**Figure 1:**
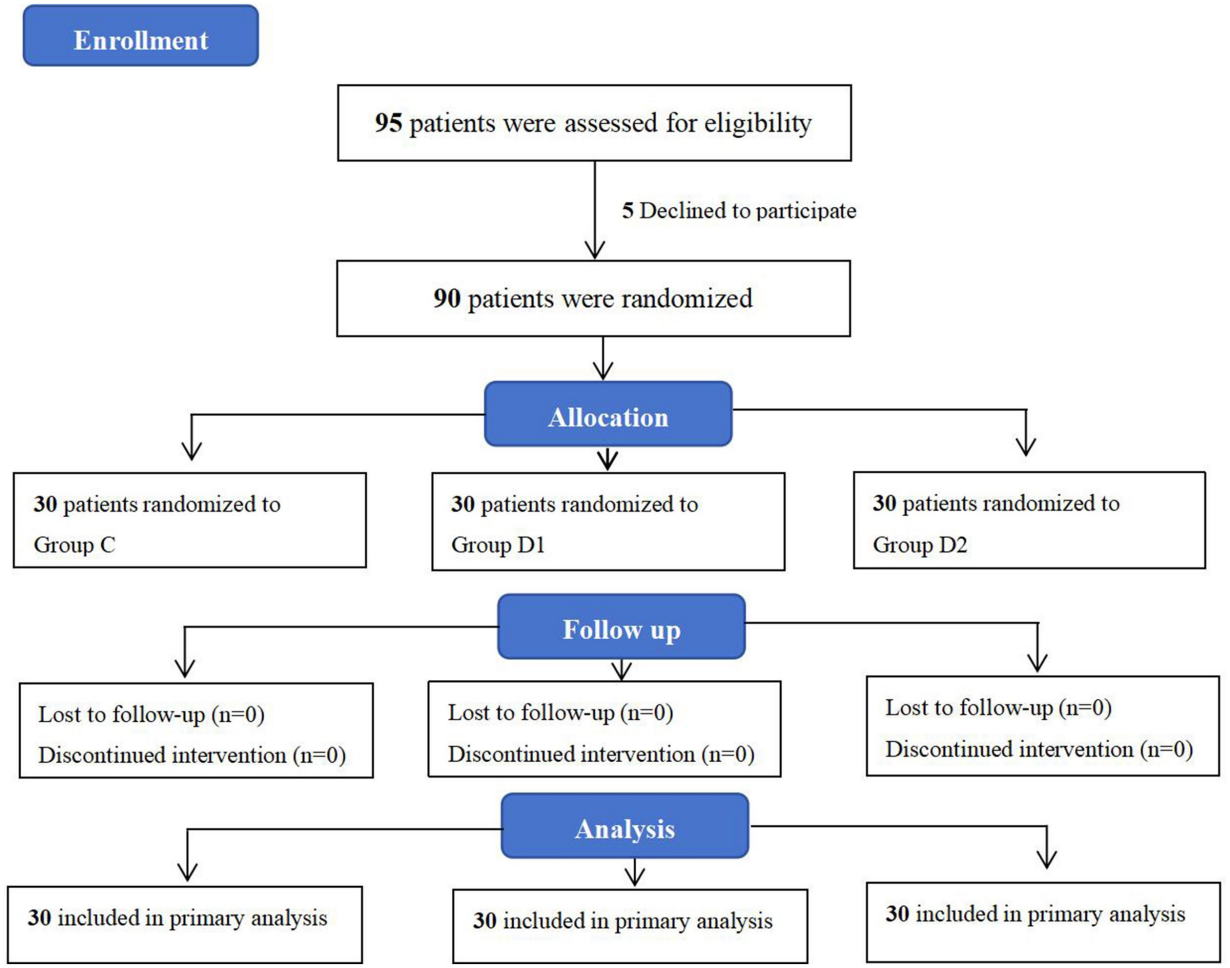
CONSORT flow chart illustrating patient selection and inclusion.

Between March 6, 2024, and September 20, 2024, a total of 95 patients scheduled for cesarean section at the People’s Hospital of Ningxia Hui Autonomous Region were enrolled. Among them, five patients declined to participate in the study. Consequently, data from 90 patients were ultimately included for statistical analysis, with 30 patients assigned to each of the three groups: C, D1, and D2 (Figure 1). The primary analysis was conducted using the intention-to-treat (ITT) principle. Patient demographic characteristics were summarized in Table 1. All patients underwent successful ultrasound-guided identification of the transversus abdominis fascial plane (21), with no procedural complications reported during the block administration.

**Table 1:** Baseline characteristics of patients.

	Group C (*n* = 30)	Group D1 (*n* = 30)	Group D2 (*n* = 30)
Age (years)	29.5 (27.0–31.0)	31.0 (29.0–33.0)	29.0 (28.0–30.0)
Weight (kg)	75.8 (70.0–84.3)	77.0 (71.8–81.3)	74.0 (70.8–78.5)
Height (cm)	162.2 (4.8)	162.1 (5.4)	162.1 (4.2)
Number of primiparas	15 (50.0%)	13 (43.3%)	13 (43.3%)

Data are presented as mean (SD), median (IQR), or n (%). Group C, 8 mg + dexamethasone 0.375% ropivacaine; Group D1, 0.5 μg/kg dexmedetomidine + 8 mg dexamethasone + 0.375% ropivacaine; Group D2, 1 μg/kg dexmedetomidine + 8 mg dexamethasone + 0.375% ropivacaine; SD, standard deviation; IQR, interquartile range.

### Evaluation of postoperative analgesic effect

#### VAS scores at various time points after cesarean section

VAS scores revealed divergent trends between rest and dynamic pain conditions. At rest, no significant differences in VAS scores were observed among groups (C, D1, D2) at 6, 12, 24, or 48 h postoperatively (*p* > 0.05 for all comparisons, Table 2).

**Table 2:** Comparison of VAS scores at different time points postoperatively.

	Time (h)	Group C (*n* = 30)	Group D1 (*n* = 30)	Group D2 (*n* = 30)	*P*	Median difference (95% CI)		
						C vs. D1	C vs. D2	D1 vs. D2
Rest pain score (VAS, 0-10 cm)	6	0.00 (0.00–0.00)	0.00 (0.00–0.00)	0.00 (0.00–0.00)	0.67	0.00 (0.00–0.00)	0.00 (0.00–0.00)	0.00 (0.00–0.00)
	12	1.00 (1.00–1.00)	1.00 (0.00–2.00)	1.00 (0.0–1.25)	0.26	0.00 (0.00–1.00)	0.00 (0.00–1.00)	0.00 (0.00–1.00)
	24	2.00 (1.00–2.25)	1.50 (1.00–2.00)	1.50 (1.00–2.00)	0.38	0.00 (0.00–1.00)	0.00 (0.00–1.00)	0.00 (0.00–0.00)
	48	1.00 (1.00–2.00)	1.0 (0.75–1.25)	1.00 (1.00–1.25)	0.19	0.00 (0.00–1.00)	0.00 (0.00–1.00)	0.00 (0.00–0.00)
Dynamic pain score (VAS, 0-10 cm)	6	0.00 (0.00–1.00)	0.00 (0.00–1.00)	0.00 (0.00–1.00)	0.78	0.00 (0.00–0.00)	0.00 (0.00–0.00)	0.00 (0.00–0.00)
	12	3.00 (2.00–4.00)	1.00 (1.00–3.00)^*^	2.00 (1.00–3.00)^*^	<0.001	1.00 (1.00–2.00)	1.00 (0.00–2.00)	0.00 (−1.00–0.00)
	24	4.00 (3.00–4.00)	3.00 (2.00–3.00)^*^	2.00 (2.00–3.00)^*^	<0.001	1.00 (0.00–1.00)	1.00 (1.00–2.00)	0.00 (0.00–1.00)
	48	3.00 (2.00–3.00)	2.00 (1.00–2.00)^*^	1.00 (1.00–2.00)^*^	<0.001	1.00 (0.00–1.00)	1.00 (1.00–1.00)	0.00 (0.00–1.00)

Data are expressed as median (IQR), and compared using Kruskal-Wallis nonparametric tests and Mann–Whitney tests. Median differences (and 95% CI) were calculated with Hodges-Lehmann estimators. All tests were two-tailed. Statistical significance was defined as *p* < 0.05. Compared with Group C, ^*^p < 0.05/3. Group C, 8 mg dexamethasone + 0.375% ropivacaine; Group D1, 0.5 μg/kg dexmedetomidine + 8 mg dexamethasone + 0.375% ropivacaine; Group D2, 1 μg/kg dexmedetomidine + 8 mg dexamethasone + 0.375% ropivacaine; CI, confidence interval; IQR, interquartile range.

Dynamic pain scores, however, differed significantly across groups. At 12 h, Group C demonstrated higher scores [median, 3.00 (IQR, 2.00–4.00)] compared with Group D1 [1.00 (1.00–3.00); median difference, 1.00 (95% CI, 1.00–2.00); *p* = 0.001] and Group D2 [2.00 (1.00–3.00); difference, 1.00 (0.00–2.00); *p* = 0.003]. By 24 h, Group C maintained elevated scores [4.00 (3.00–4.00)], with significant differences vs. Group D1 [3.00 (2.00–3.00); difference, 1.00 (0.00–1.00); *p* < 0.001] and Group D2 [2.00 (2.00–3.00); difference, 1.00 (1.00–2.00); *p* = 0.009]. At 48 h, Group D2 exhibited the lowest dynamic pain scores [1.00 (1.00–2.00)], differing significantly from Group C [3.00 (2.00–3.00); difference, 1.00 (1.00–1.00); *p* = 0.001]. No significant differences were observed between Groups D1 and D2 at any time point (*p* > 0.05) (Table 2).

#### The incidence rate of moderate to severe pain at various time points following cesarean section

Overall, the incidence of moderate to severe resting pain was low across all groups at all-time points, with no significant differences observed between groups. For dynamic pain, significant intergroup differences were noted at 12 h postoperatively (26.7% in Group C, 13.3% in D1, and 3.3% in D2; *p* = 0.04). By 24 h, dynamic pain incidence peaked in Group C (53.3%, 16/30), followed by D1 (13.3%, 4/30) and D2 (10.0%, 3/30). Compared with Group C, both D1 and D2 exhibited statistically significant risk reductions (D1: risk ratio = 7.43, 95% CI 2.08–26.55, *p* = 0.001; D2: risk ratio = 10.29, 95% CI 2.56–41.37, *p* = 0.002). At 48 h, dynamic pain incidence decreased to 13.3% (4/30) in Group C, 3.3% (1/30) in D1, and 0.0% in D2, with no significant differences between groups (Table 3).

**Table 3:** Comparison of the incidence of moderate to severe pain at various time points postoperatively.

	Time (h)	Group C (*n* = 30)	GroupD1 (*n* = 30)	Group D2 (*n* = 30)	*P*	Risk ratio difference (95% CI)		
						C vs. D1	C vs. D2	D1 vs. D2
Rest pain	6	0 (0.0%)	0 (0.0%)	0 (0.0%)	–	–	–	–
	12	2 (6.7%)	1 (3.3%)	0 (0.0%)	0.77	2.07 (0.18–24.15)	0.93 (0.85–1.03)	0.97 (0.91–1.03)
	24	2 (6.7%)	3 (10.0%)	1 (3.3%)	0.87	0.64 (0.10–4.15)	2.07 (0.18–24.15)	3.22 (0.89–32.89)
	48	0 (0.0%)	0 (0.0%)	1 (3.3%)	1	-	1.03 (0.97–1.11)	1.03 (0.97–1.11)
Dynamic pain	6	0 (0.0%)	1 (3.3%)	0 (0.0%)	1	1.03 (0.97–1.11)	-	0.97 (0.91–1.03)
	12	8 (26.7%)	4 (13.3%)	1 (3.3%)	0.04	2.36 (0.63–8.92)	10.55 (1.23–90.66)	4.46 (0.47–42.51)
	24	16 (53.3%)	4 (13.3%)^*^	3 (10.0%)^*^	<0.001	7.43 (2.08–26.55)	10.29 (2.56–41.37)	1.39 (0.28–6.80)
	48	4 (13.3%)	1 (3.3%)	0 (0.0%)	0.122	4.46 (0.47–42.51)	0.87 (0.75–1.00)	0.97 (0.91–1.03)

Data are presented as n (%), and compared using Fisher exact test. All tests were two-tailed. *P* < 0.05 was considered to indicate a statistically significant difference. Compared with Group C, ^*^p < 0.05/3. Group C, 8 mg dexamethasone + 0.375% ropivacaine; Group D1, 0.5 μg/kg dexmedetomidine + 8 mg dexamethasone + 0.375% ropivacaine; Group D2, 1 μg/kg dexmedetomidine + 8 mg dexamethasone + 0.375% ropivacaine; CI, confidence interval.

### Secondary outcomes

Significant intergroup differences were observed in the first request of rescue analgesia within 24 h postoperatively, with 13.3% (4/30) of patients in Group C requiring analgesia compared to none in Group D1 or D2 (*p* = 0.01). From 24 to 72 h, no significant differences were detected among groups (*p* = 0.78). The duration of sensory blocked, assessed via temperature and mechanical discrimination, differed significantly across groups (*p* < 0.001). No significant differences were observed in time to gastrointestinal recovery, incidence of postoperative nausea and vomiting (PONV), or ObsQoR-10 scores across groups (*p* > 0.05) (Table 4).

**Table 4:** Comparison of secondary outcomes.

	Group C (*n* = 30)	Group D1 (*n* = 30)	Group D2 (*n* = 30)	*P*
First request of rescue analgesia, n (%)				
Within the first 24 h	4 (13.3%)	0	0	0.01
24–72 h	2 (6.7%)	1 (3.3%)	0	0.78
The duration of sensory block (h) (temperature discrimination)	62.11 (20.06)	88.05 (26.10)^*^	104.93 (20.45)^*△^	<0.001
The duration of sensory blocked (h) (mechanical discrimination)	60.62 (21.19)	83.80 (23.30)^*^	96.88 (12.61)^*△^	<0.001
Time to gastrointestina Recovery (h)	31.87 (16.23)	32.00 (20.76)	37.65 (15.80)	0.36
Incidence of PONV				
0–6 h	3 (10.0%)	4 (13.3%)	1 (3.3%)	0.34
6–24 h	5 (16.7)	3 (10.0%)	5 (16.7%)	0.68
ObsQoR-10 scales score				
1 day postoperatively	68.00 (63.00–69.25)	65.50 (60.00–69.25)	68.50 (66.00–70.00)	0.13
3 day postoperatively	81.00 (75.75–85.00)	83.50 (80.75–86.25)	84.50 (80.75–86.00)	0.07
7 day postoperatively	94.00 (93.00–95.00)	94.50 (93.00–96.00)	95.00 (93.00–96.00)	0.16

Data are presented as mean (SD), median (IQR), or n (%). Continuous variables conformed to a normal distribution were compared among the three groups using one-way analysis of variance. Kruskal-Wallis nonparametric tests was used for variables not conforming to normal distribution. All tests were two-tailed. Statistical significance was defined as *P* < 0.05. Compared with Group C, ^*^p < 0.05. Compared with Group D1, ^△^*p* < 0.05. Group C, 8 mg dexamethasone + 0.375% ropivacaine; Group D1, 0.5 μg/kg dexmedetomidine + 8 mg dexamethasone + 0.375% ropivacaine; Group D2, 1 μg/kg dexmedetomidine + 8 mg dexamethasone + 0.375% ropivacaine; CI, confidence interval; IQR, interquartile range.

## Discussion

This study demonstrated that combining varying doses of dexmedetomidine with dexamethasone in transversus abdominis plane block (TAPB) significantly improved movement-associated VAS scores within 48 h post-cesarean and reduced moderate-to-severe pain incidence in the first 24 h. Specifically, 1 μg/kg dexmedetomidine combined with 8 mg dexamethasone in TAPB prolonged sensory block duration up to 4 days without requiring rescue analgesia.

Effective postoperative analgesia remains a critical concern (22). However, the results from the control group in this study demonstrated that using dexamethasone alone for TAPB was associated with a high incidence (53%) of moderate-to-severe pain during the first 24 h postoperatively, indicating that this analgesic regimen remains inadequate. This is similar to the results of many previous studies, where despite the use of different postoperative analgesics and pain management strategies, the incidence of pain after cesarean section remains high, ranging from 25.5 to 80% (23–25). Overall, women who have undergone cesarean section still do not receive adequate analgesic treatment.

Despite literature supporting the role of quadratus lumborum block (QLB) in improving postoperative pain following cesarean section, the analgesic efficacy of TAPB has also been widely confirmed (26, 27). Current comparative studies indicate no significant difference between QLB and TAPB in enhancing postoperative analgesic efficacy following cesarean delivery (28). From a practical standpoint, TAPB, as a routine technique for post-cesarean analgesia, offers advantages over QLB in terms of easier mastery of the puncture technique and lower associated risks. This motivated our further exploration of strategies to optimize TAPB.

Our trial explored a multimodal approach by combining dexmedetomidine (α2-agonist) and dexamethasone (anti-inflammatory) as adjuvants to ropivacaine TAPB. The main results of the trial indicate that the use of dexmedetomidine at varying doses combined with dexamethasone to ropivacaine-based TAPB can significantly reduce the dynamic VAS scores at 12, 24, and 48 h postoperatively in cesarean section patients, as well as decrease the incidence of moderate to severe dynamic pain within the first 12 and 24 h postoperatively. These findings indicate that the dexmedetomidine-dexamethasone-ropivacaine combination can alleviate postoperative movement-related pain in post-cesarean section patients and significantly reduce the incidence of moderate to severe pain during movement after surgery. Notably, the absence of rescue analgesia requests in both D1 and D2 groups within the first 24 h suggests that combination of dexmedetomidine with dexamethasone as an adjunct to ropivacaine for TAPB may reduce opioid reliance, aligning with enhanced recovery after surgery (ERAS) principles. This synergistic effect likely arises from the distinct mechanisms of the adjuvants (13, 29): dexmedetomidine inhibits nociceptive signaling via spinal and periphera α2-adrenergic receptors, while its vasoconstrictive properties may prolong ropivacaine’s local action by delaying systemic absorption. Concurrently, dexamethasone’s anti-inflammatory effects may attenuate surgical site edema, amplifying analgesic benefits. Additionally, our study results also suggest that the combination of dexmedetomidine with dexamethasone as an adjunct to TAPB can significantly prolong the duration of postoperative sensory blocked, with an average block duration of about 4 days, which is similar to the findings of the study by Herman et al. (30). However, Aliste et al. (18) in their study, when using dexamethasone combined with dexmedetomidine as an adjunct to brachial plexus block, found the motor block time, sensory block time, and analgesic time to be 21.5, 21.6, and 25.5 h, respectively. These findings differ somewhat from our experimental results, which may be related to differences in dosage, timing of administration, the nerves affected, and the site of origin of the nociceptive stimuli.

Given that this study involves the maternal and infant population, it is essential to pay attention to the safety and rationality of medication use. While chronic glucocorticoid use is linked to maternal hyperglycemia and infection risks, single-dose dexamethasone administration has not shown these adverse effects (31). Low molecular weight drugs (<200 Da) readily transfer into breast milk (32); however, dexamethasone (molecular weight: 392.4 Da) is unlikely to be ingested by infants during breastfeeding. Gyamfi-Bannerman et al. (33) further demonstrated that antenatal corticosteroids do not impair neonatal neurodevelopment. In this trial, no maternal or neonatal adverse outcomes were observed with dexamethasone. Additionally, 8 mg dexamethasone as a peripheral nerve block adjuvant has been validated as neurologically safe (34, 35), justifying our dose selection. Similarly, dexmedetomidine has shown no neurological complications in peripheral nerve blocks (36, 37). Studies have assessed the concentration of dexmedetomidine transfer into breast milk following its use during cesarean section and found that the drug concentration in breast milk is very low, suggesting that it is unlikely to cause harm to infants who are breastfed (38, 39). Our findings align with these reports, supporting its safety as a TAPB adjuvant.

The limitations of this study include the following issues. Firstly, this study did not measure the plasma concentrations of dexamethasone and other related drugs in maternal blood and breast milk, which precludes the assessment of any potential correlation between the use of these medications and infant safety. However, no adverse effects related to these drugs were observed during the course of this trial. Secondly, the data in this study were based on subjective self-assessments reported by the patients, but the randomized grouping could mitigate its impact on the results. Thirdly, the results from the control group in this study suggest that even with a multimodal analgesic regimen including intrathecal opioids, TAPB, and PCA with opioid drugs, the incidence of moderate to severe pain within the first 24 h after cesarean section remains high. This indicates that achieving ideal analgesic effects after cesarean section is challenging, hence the study could not forgo the combined use of a PCA pump containing opioid drugs during the process. Fourthly, due to equipment limitations, this study utilized disposable mechanical infusion pumps lacking electronic monitoring functionality. Consequently, the total PCA consumption for each group could not be recorded. As a pre-specified secondary outcome measure, total PCA consumption would have facilitated a more detailed evaluation of analgesic efficacy. Theoretically, acquisition of this data could have rendered the assessment of the analgesic regimens in this study more comprehensive. Fortunately, the analgesic regimen in the experimental group of this study achieved the desired analgesic effect after cesarean section. In the future, it will be necessary to build upon this foundation to explore further optimization of the dose of opioid drugs in the PCA pump when using dexmedetomidine combined with dexamethasone as an adjunct to TAPB.

In summary, the combination of dexmedetomidine with dexamethasone as an adjunct to TAPB can significantly improve the degree of postoperative pain and the incidence of moderate to severe pain after cesarean section, enhancing the quality of postoperative recovery for parturients. Additionally, our study results also revealed that this combined medication as an adjunct to TAPB can significantly prolong the duration of postoperative sensory block, with an average block duration of approximately 4 days.

## Data Availability

The raw data supporting the conclusions of this article will be made available by the authors, without undue reservation.
